# A striking response of plasmablastic lymphoma of the oral cavity to bortezomib: a case report

**DOI:** 10.1186/s40364-015-0053-0

**Published:** 2015-11-04

**Authors:** Makoto Hirosawa, Hiroaki Morimoto, Ryo Shibuya, Shohei Shimajiri, Junichi Tsukada

**Affiliations:** Hematology, University of Occupational and Environmental Health, 1-1 Iseigaoka, Yahatanishi-ku, Kitakyushu, 807-8556 Japan; Department of Pathology and Oncology, University of Occupational and Environmental Health, Kitakyushu, Japan; Department of Pathology and Cell Biology, University of Occupational and Environmental Health, Kitakyushu, Japan

**Keywords:** Bortezomib, Plasmablastic lymphoma, Oral cavity

## Abstract

**Background:**

Plasmablastic lymphoma (PBL) is a rare and aggressive subtype of non-Hodgkin diffuse large B-cell lymphoma originally with a predilection to the oral cavity of patients infected with HIV. However, PBL of extraoral sites possesses clinicopathological characteristics distinct from oral PBL. Recently, therapeutic approaches using a proteasome inhibitor bortezomib to PBL of extraoral sites have been reported. We present a PBL patient with a bulky tumor of the oral cavity, who dramatically responded to bortezomib.

**Case Presentation:**

The patient was a 58 year-old Japanese male, who presented with a rapidly progressive history of a swelling on his left cheek and restricted mouth opening. He did not have a history or evidence of immunosuppression including HIV infection. A computed tomography demonstrated a bulky tumor in the oral cavity without enlarged lymph nodes. The tumor showed the proliferation of large lymphoid cells with centroblastic morphology, which were positive for CD138, CD38, CD56 and MUM-1, and negative for CD20, CD79a, BCL-6 and HHV8. The Ki-67 proliferation index was almost 100 %. Neither osteolytic lesions nor M-protein was observed. One week after the initiation of bortezomib, a marked regression of the oral tumor was obtained.

**Conclusions:**

Thus, our case demonstrated the effectiveness of bortezomib on PBL of the oral cavity as well as the extraoral sites.

## Background

Plasmablastic lymphoma (PBL) was originally described in 1997 by Delecluse et al., as an aggressive variant of large B-cell lymphoma with plasmablastic features occurs in the oral cavity or in the jaw of human immunodeficiency virus (HIV)-infected patients [1]. PBL has been initially seen in the clinical setting of HIV infection. The tumor cells are large lymphoid cells with centroblastic/immunoblastic features, which typically express plasma cell markers such as CD38 and CD138, and lack expression of mature B-cell markers. This lymphoma exhibits rapid disease progression and resistance to various chemotherapy regimens [2].

However, the development of PBL in HIV-negative patients has been recently reported [3]. When compared with HIV-positive PBL, HIV-negative PBL tends to occur in extraoral sites and morphologically shows a more apparent plasma cell differentiation [4–6].

Because of the rarity and aggressiveness of the disease, no standard chemotherapy for PBL has been established. In this regard, therapeutic approach with bortezomib to HIV-negative PBL [7–9] and HIV-positive PBL [9–11] has been recently reported. However, primary sites of involvement in these PBL patients treated with bortezomib were extraoral. We herein report a case with PBL of the oral cavity morphologically showing centroblastic features as reported by Delecluse et al. [1]. Bortezomib induced a dramatic regression of a bulky tumor in the oral cavity of the patient.

## Case presentation

**Fig. 1:**
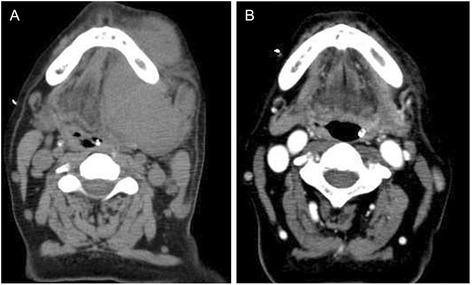
Transverse CT scan of the oral cavity showing an oral tumor at diagnosis (**a**) and 30 days after the initiation of bortezomib treatment (**b**)

**Fig. 2:**
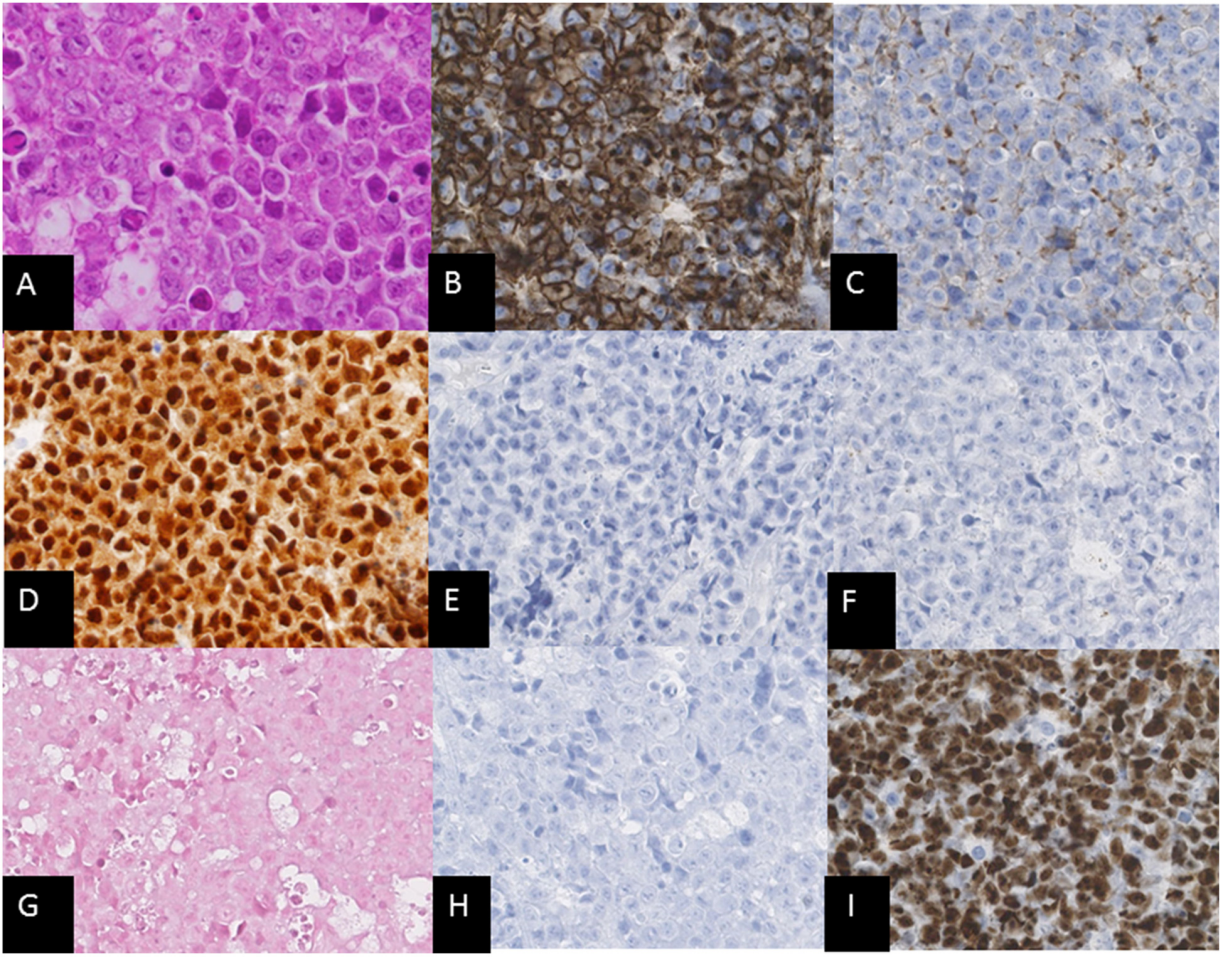
**a** Hematoxylin and eosin staining showing large lymphoid cells with centroblastic morphology (X400). Cells of the oral tumor (X200) were immunohistologically positive for **b** CD138 and **c** CD38, **d** MUM-1, and were negative for **e** CD20, **f** CD79a, **g** EBV-encoded RNA *in situ* hybridization (EBER-ISH) and **h** HHV8. **i** The Ki-67 proliferation index was almost 100 %

The patient is a 58 year-old Japanese male presented with a rapidly progressive history of a swelling on his left cheek and restricted mouth opening. He had no history of HIV infection, multiple myeloma or underlying immunosuppression. Physical examination showed a bleeding tumor of the oral cavity. The laboratory findings on admission were: hemoglobin 10.9 g/dL, white blood cell count 11.4×10^9^/L, platelet 197×10^9^/L serum lactate dehydrogenase (LDH) 2232 U/L (normal value: 119–229), serum creatinine 1.68 mg/dL (normal value: 0.6–1.1), serum uric acid 12.7 mg/dL (normal value: 3.6–8.0), serum potassium 4.9 mg/dL (normal value: 3.6–4.9), serum phosphorus 6.2 mg/dL (normal value: 2.5–4.7), serum calcium 11.3 mg/dL (normal value: 8.7–10.3). Serum IgG, IgM, and IgA levels were within the normal range. Serum protein electrophoresis had no M-spike. Serology for HIV was negative. There were no osteolytic lesions on bone survey. A computed tomography (CT) demonstrated a bulky tumor localized in the mucosa of the oral cavity (Fig. 1a), without enlargement of the systemic lymph nodes. Biopsy of the oral mass showed diffuse proliferation of large lymphoid cells (Fig. 2a) with centroblastic morphology, which were positive for CD138 (Fig. 2b), CD38 (Fig. 2c), CD56, and MUM-1 (Fig. 2d) and negative for CD20 (Fig. 2e), CD79a (Fig. 2f), CD3 and BCL-6. EBV-encoded RNA *in situ* hybridization (EBER-ISH) (Fig. 2g) and HHV8 (Fig. 2h) were negative. The Ki-67 proliferation index was almost 100 % (Fig. 2i). No apparent plasmacytic differentiation of the tumor cells was observed. G-banding analysis of the tumor showed abnormal karyotypes: 46, X, −Y, dup(1)(q42q21), −6, +7, +7, −8, add(14)(q32), add(20)(p11.2), +mar/ 82<3n>, XX,-Y, dup(1)×2, −4, +5, +6, +6, +7, +7, +7, +7, +7, −8, −8, +9, +9, +11, add(14)×2, +15, +5mar. Although no lymphoma cells were found in his peripheral blood, bone marrow examination showed infiltration of large atypical lymphoid cells, suggesting bone marrow involvement. The patient was diagnosed with stage IVA PBL.

Immediately after diagnosis, the patient was treated with THP-COP composed of pirarubicin, cyclophosphamide, vincristine and prednisolone. Since pirarubicin (THP) has been reported to have comparable efficacy to doxorubicin with a lower incidence of cardiac toxicity in aggressive non-Hodgkin lymphoma treatment, THP-COP therapy was used [12]. Intravenous hydration and allopurinol also started.

Despite the prompt treatment, the oral tumor continued to enlarge, with increasing risk of suffocation. Therefore, bortezomib was started on day 9 of admission. Bortezomib was given at a dose of 1.3 mg/m^2^ on days 1, 8 and 15 without dexamethasone in the first cycle. In the second cycle, the patient was treated with bortezomib in combination with dexamethasone. One week after the initiation of bortezomib, a marked regression of the oral tumor was observed, and one month after its initiation, no significant swelling of the oral cavity was detected (Fig. 1b). Serum LDH levels also rapidly improved. However, since resistance to bortezomib developed after the third cycle of bortezomib, the patient was salvaged with EPOCH and irradiation. He had disease-progression and died five months post-diagnosis.

## Discussion

The original report by Delecluse et al. described a series of 16 patients with PBL [1]. They all presented with the involvement of the oral cavity. 15 of the 16 PBL patients were infected with HIV. Resistance of PBL to various chemotherapy regimens even in the multidrug setting or combined with radiotherapy has been shown. The treatment outcome for PBL generally remains poor with a short survival [2].

The occurrence of PBL in HIV-negative individuals has been also recognized [3]. Although HIV-negative PBL has been initially thought to be associated with an underlying immunosuppressive state including organ transplantation, autoimmune diseases and lymphoproliferative diseases, no significant immunodeficiency was found in other cases [3, 6]. In addition, comparative studies between HIV-positive and HIV-negative PBL have been reported [4–6]. When compared with HIV-negative PBL, PBL of the oral cavity tends to occur in HIV-positive patients and shows morphological features of centroblasts or immunoblasts without plasmacytic differentiation, as originally described by Delecluse et al. [1]. On the other hand, extraoral PBL appears to occur in HIV-negative patients morphologically with a more apparent plasma cell differentiation. Thus, clinicopathological differences between oral PBL and extraoral PBL have been pointed out.

Several studies have reported PBL cases treated with a proteosome inhibitor bortezomib [7–11]. Bose et al. showed a HIV-positive patient with PBL of the extraoral sites, who had dramatic disease regression following only two doses of bortezomib (days 1 and 4) [10]. A rapid improvement of HIV-negative PBL tumor infiltrating the left gluteus and erector spinae muscles after the first cycle of BD therapy (bortezomib plus dexamethasone) was also reported [7]. Tumor lysis syndrome was induced by bortezomib combined with rituximab, cyclophosphamide and dexamethasone (R-CBortP) in a patient with HIV-negative PBL of the extraoral sites, 24–48 h after the first injection of bortezomib [8]. Moreover, early disease relapses have been observed despite the initial response to bortezomib. In this regard, a study using three PBL patients (two HIV-positive and one HIV-negative) demonstrated a durable response of PBL to a combination of bortezomib with dose-adjusted EPOCH with the disease-free survival ranged from 12 to 24 months [9]. However, in these previous reports using bortezomib, the primary sites of PBL involvement were extraoral.

Carbone et al. reported that HIV-associated PBL consistently shows the BCL-6 negative/IRF4 (MUM-1) positive/CD138 (Syn-1) positive phenotype, and therefore reflects post-germinal center (GC) B-cells in all cases examined [13]. They pointed out that post-GC cells undergoing maturation toward plasma cells switch off BCL-6 expression. In this regard, the original report by Delecluse et al. showed that only a weak and partial expression of BCL-6 was detected in 4 of 14 tested cases, whereas the remaining cases were negative for BCL-6 [1]. Activated B-cell-like (ABC) diffuse large B-cell lymphoma (DLBCL), arises from post-GC B-cell, is characterized by constitutive activation of the NF-KB pathway. Bortezomib has been demonstrated to enhance the activity of chemotherapy in ABC DLBCL, but not in GC DLBCL [14]. These findings show a potential benefit of bortezomib in treatment for PBL as well as for ABC DLBCL. However, they further pointed out that bortezomib was inactive as a single agent, suggesting that tumor growth is regulated by additional anti-apoptotic signals unaffected by bortezomib [14].

Thus, getting a better understanding of the unique features of PBL is essential. Our report demonstrates therapeutic benefit of bortezomib for PBL of the oral cavity as well as extraoral PBL.

## Conclusion

PBL is an aggressive variant of large B-cell lymphoma, originally with a predilection to the oral cavity of patients infected with HIV. Herein, our report presents with the first case of PBL of the oral cavity, who showed a drastic response to bortezomib. Symptoms of the patient immediately disappeared following bortezomib administration. The tumor regression was further obtained without cytotoxic chemotherapy.

## Consent

Because the patient has died and his kin are not traceable, the ethics committee of our institute, University of Occupational and Environmental Health, Japan has approved publication of this report.

## Abbreviations

ABC: Activated B-cell-like
CT: Computed tomography
DLBCL: Diffuse large B-cell lymphoma
EBER-ISH: EBV-encoded RNA *in situ* hybridization
GC: Germinal center
HIV: Human immunodeficiency virus
LDH: Lactate dehydrogenase
OS: Overall survival
PBL: Plasmablastic lymphoma
